# A wireless spinal stimulation system for ventral activation of the rat cervical spinal cord

**DOI:** 10.1038/s41598-021-94047-1

**Published:** 2021-07-21

**Authors:** Matthew K. Hogan, Sean M. Barber, Zhoulyu Rao, Bethany R. Kondiles, Meng Huang, William J. Steele, Cunjiang Yu, Philip J. Horner

**Affiliations:** 1 Department of Neurosurgery, Center for Neuroregeneration, Houston Methodist Research Institute, Houston, USA; 2 University of Houston, Houston, USA; 3 International Collaboration on Repair Discovories, University of British Columbia, Vancouver, Canada

**Keywords:** Regeneration and repair in the nervous system, Spinal cord injury, Regenerative medicine, Biomedical engineering

## Abstract

Electrical stimulation of the cervical spinal cord is gaining traction as a therapy following spinal cord injury; however, it is difficult to target the cervical motor region in a rodent using a non-penetrating stimulus compared with direct placement of intraspinal wire electrodes. Penetrating wire electrodes have been explored in rodent and pig models and, while they have proven beneficial in the injured spinal cord, the negative aspects of spinal parenchymal penetration (e.g., gliosis, neural tissue damage, and obdurate inflammation) are of concern when considering therapeutic potential. We therefore designed a novel approach for epidural stimulation of the rat spinal cord using a wireless stimulation system and ventral electrode array. Our approach allowed for preservation of mobility following surgery and was suitable for long term stimulation strategies in awake, freely functioning animals. Further, electrophysiology mapping of the ventral spinal cord revealed the ventral approach was suitable to target muscle groups of the rat forelimb and, at a single electrode lead position, different stimulation protocols could be applied to achieve unique activation patterns of the muscles of the forelimb.

## Introduction

Paralysis of the hand is a common condition following cervical spinal cord injury (SCI), brain trauma, or stroke^1^. Hand and arm recovery is the top ranked treatment priority for those suffering from cervical SCI^2,3^, thus interventions to target hand and arm recovery are of critical importance. Yet, they have garnered notably less attention compared to interventions that target lower limb functions, such as standing and walking. The concept of electrical stimulation as a potential therapy for central nervous system (CNS) trauma has gained traction in recent years. However, despite documented improvements in functional recovery from a combination of rehabilitation with neural stimulation, rehabilitation alone remains the only therapy of standard practice for chronic neural injury^4–7^.

Still, electrical stimulation has been demonstrated to effect persistent changes in connectivity and synaptic strength of stimulated circuits in experimental models of the intact and injured CNS. Work by Seeman et. al. determined that paired electrical pulses between two regions of the non-human primate sensorimotor cortex were able to facilitate enhanced synaptic strength between the stimulated regions in a manner consistent with spike time dependent plasticity (STDP), indicating that electrical stimulation can strengthen connections in the brain^8^. Following injury to one side of the corticospinal tract (CST), Carmel et al. showed that stimulating the contralateral motor cortex facilitated an improvement in skilled paw placement, which persisted beyond the period of stimulation and was accompanied by sprouting of stimulated CST fibers into the contralateral denervated region^9^. Further, electrical stimulation has been implicated in enhanced axonal outgrowth both in vitro and in vivo following SCI^10–13^.

**Figure 1:**
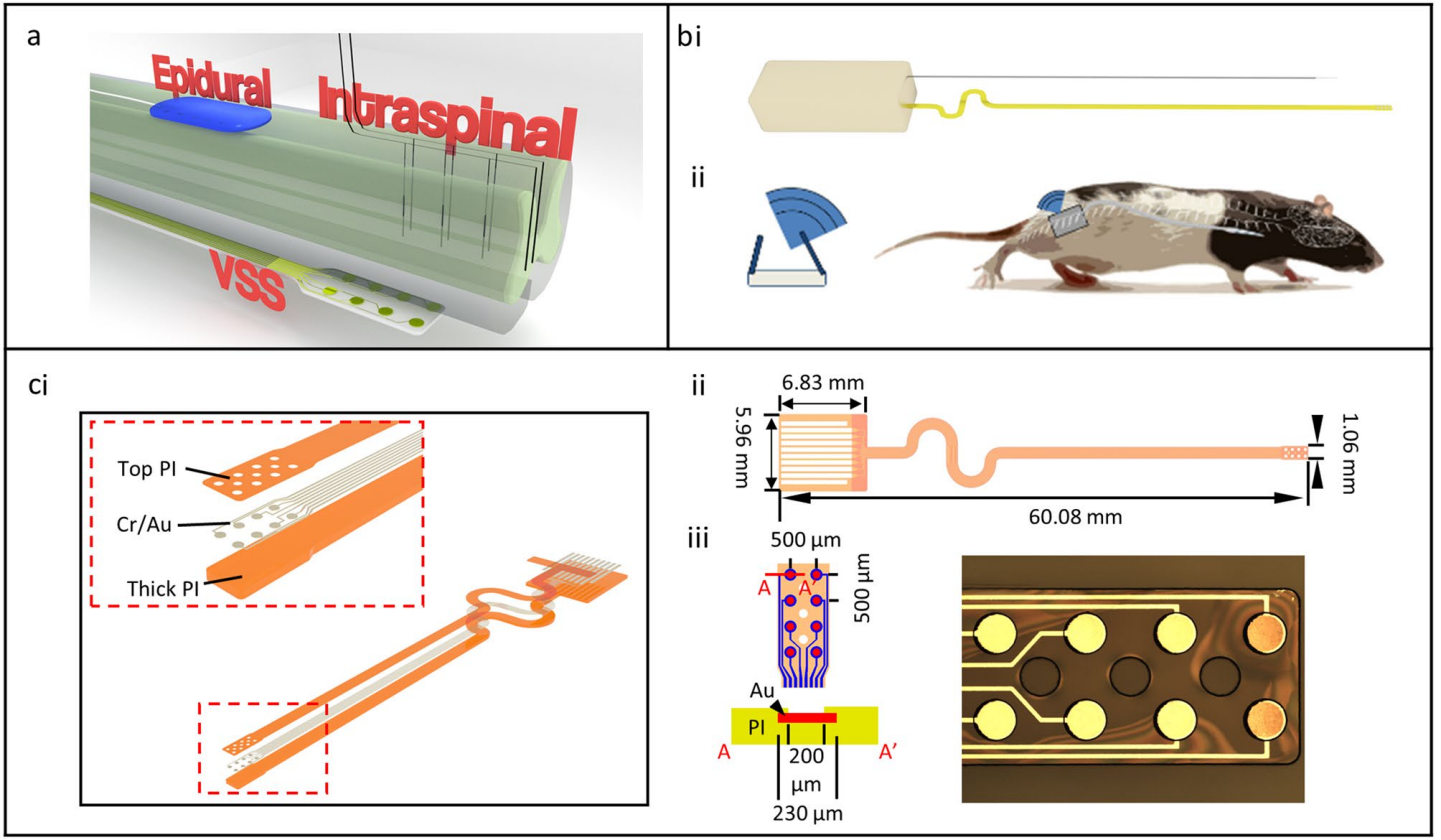
Device and electrode design. (**a**) Typically, spinal stimulation is performed via traditional epidural dorsal spinal electrodes or via penetrating intraspinal electrodes. We have developed a ventral spinal stimulation (VSS) approach that targets the ventral portion of the spinal cord and is geometrically closer to motor associated circuitry. (**b**) We (i) generated a spinal stimulator with wireless inductive charging fitted with a low bending stiffness biocompatible electrode developed using soft electronics fabrication techniques and (ii) designed the device to be implanted into the ventral epidural space and controlled wirelessly with no percutaneous wires. (**c**) illustrates (i) the exploded schematic of the electrode (the inset is the detail of the exposed stimulating part), (ii) the dimensions of the electrode system, and (iii) a brightfield microscope image of a finished electrode tip.

Electrical stimulation may provide critical benefit to upstream and downstream targets following CNS injury. Two common modes of spinal stimulation are intraspinal microstimulation (ISMS) and dorsal epidural stimulation (Fig. 1). While non-invasive techniques such as transcranial magnetic stimulation (TMS) and non-invasive electrical stimulation have demonstrated benefit, both techniques evoke larger current fields and lack potential spatial targeting characteristic of more invasive approaches. Both ISMS and epidural stimulation have proven beneficial in the context of cervical injury in rats. In the case of ISMS, work from the Moritz and Horner labs demonstrates therapeutic benefits in a rodent cervical hemicontusion model^4,14^. Stimulation of spinal motor pools associated with the circuits of the forelimb and hand significantly improved digit extension, pronation, and supination of the wrist in a forelimb reaching tasks in rats, and the functional improvement persisted beyond the therapeutic stimulation window^15,16^. Further, work from the Edgerton lab demonstrated dorsal epidural spinal stimulation (DSS) at the injured cervical level reduced aberrant co-activation of antagonistic muscle groups in rats^17^.

Both ISMS and DSS have proven beneficial in the context of SCI; still, each method has drawbacks that limit therapeutic potential. ISMS techniques allow for precise targeting of motor circuitry in the spinal cord. Intraparenchymal wires can be guided to discrete spinal segments and lamina to stimulate select motor neuron populations^4,18^. The relative precision of implantation and proximity to the ISMS target allows for a more controlled modulation of activity in the spinal cord compared with surface electrical current generated by DSS. Indeed, a study by Sharpe and Jackson in monkeys demonstrated that ISMS produced a higher degree of arm/forearm/hand selectivity at lower thresholds of cervical stimulation when compared with DSS^19^. Further, a direct comparison of the thresholds of activation demonstrated that ISMS approaches achieved significant activation of muscles at lower currents when compared with epidural DSS. Given the enhanced spatial targeting of the ISMS approach, there may be a significant clinical benefit of intraspinal stimulation, and research has extended beyond the cat and rodent model into larger animal models. Specifically, mechanical testing of ISMS approaches has been performed in the domestic pig^20^. Ultrasound technology was used to guide probes into the spinal cord with preliminary functional tests of motor stimulation^21^. Further, the feasibility and mapping of spinal networks was studied in the lumbosacral enlargement in non-human primates^22^. Ultimately, however, these studies are preliminary, and functional recovery studies have yet to be performed in species other than rats and cats. While ISMS has not yet been clinically applied for limb movement restoration in humans, there are reports of intraspinal approaches employed to improve micturition in paraplegic patients^23,24^. Still, a major barrier for ISMS translation is that penetrating electrodes may cause local scarring; however, it is possible scarring could be minimized through proper material choice and surgical approach^25^. In contrast, DSS has been widely applied in the human clinical setting for chronic neuropathic pain syndromes with a favorable morbidity profile^26^. Therefore, DSS has appeal given the less invasive placement and translational potential relative to ISMS. Still, motor neurons are located within the ventral horn of the spinal gray matter; and, as such, DSS is less likely to activate motor pools without recruiting segmental circuits. Further, while scarring occurs using an ISMS approach, there has been no demonstration that currently employed approaches fail, only that thresholds of activation rise over time^27^. It is likely that DSS functions by activating central pattern generators either directly or through indirect activation of sensory or propriospinal pathways^28,29^. As such, direct activation of motor neuron circuitry may be less feasible with this approach. Direct activation or recording of cervical motor neuron activity may have unique or complementary applications in the development of spinal computer interfaces, relays for brain machine interfaces, or exoskeletons as a diagnostic of motor circuit connectivity and functionality in experimental models and in humans with SCI.

Ventral epidural spinal stimulation (VSS) has not been extensively studied, likely due to the surgical challenge of accessing the ventral epidural space in a small animal. However, the ventral epidural approach has been considered to encourage inspiratory muscle pacing in dogs via high frequency stimulation of the ventral thoracic spine^30^. Further, Sharpe and Jackson posit that VSS activates unique circuitry when compared with dorsal stimulation in monkeys^19^, and so there may be distinct clinical implications for VSS when compared with DSS. While there is significant challenge in placing delicate electronics in rodents due to anatomic constraints, use of the ventral surgical approach for spinal decompression and stabilization occurs in select humans cases (e.g., SCI and cervical myelopathy)^31–34^. Considerable engineering may be required to adapt existing electrode designs; however, given that surgical exposure from a ventral aspect is used clinically, there may be opportunity to develop an approach for ventral placement of electrodes in humans. We developed a precise technique for placing soft electronics in the ventral epidural space in rodents using a dorsal surgical approach that could potentially be adapted clinically for more typical spinal surgeries such as spinal decompression or stabilization. Further, data presented in this manuscript indicate that VSS may generate combined sensory root activation and spinal evoked movements similar to dorsal stimulation in rats. This was achieved by stimulating a point in the ventral space across a reference electrode. However, VSS had the unique capability to produce more discrete motor movements when stimulation was directed between adjacent electrodes on the ventral surface of the lower cervical region. Together, these data demonstrate that VSS may be a useful tool to interrogate mechanisms of upper motor neuron plasticity as well as a component of therapeutic systems to modulate motor function of the hand alone or in combination with a brain computer interface or exoskeleton.

Our aim was to develop a system with not only the feasibility and safety advantages of a dorsal epidural stimulator, but also one that retained a higher capacity to target ventrally located motor associated circuitry previously only accessible by intraspinal systems. Here, we demonstrate a stand-alone VSS system and implantation technique that minimizes collateral epidural and parenchymal scarring, while also confirming the durability and longevity of our wirelessly powered electronic system in vivo. The ventral location of the epidural electrodes allowed for experimentation with a unique current path associated with motor circuitry of the upper limb and hand.

## Results

### VSS system

We developed a spinal stimulation system designed to (1) be completely wireless and fully implantable with no percutaneous wires, allowing for freedom of movement, (2) operate up to 6 months via wireless recharging strategies for longitudinal studies, (3) communicate with a central hub for implant status monitoring and protocol adjustment, (4) unilaterally deliver stimulus to the left or right ventral aspect of the cervical spinal cord, (5) deliver fully programmable stimulus patterns with control of anodic and cathodic current amplitudes and pulse widths, interstimulation intervals (ISI), and independent and programmable selection of anode and cathode among all channels, and (6) be biocompatible and well-tolerated over the course of a study. Our system included a rechargeable 22 mA/h lithium ion battery and inductive wireless recharging based on technologies reported in the literature^35^. Measurements indicated a recharge rate ranging from 1 to 10 mW when the receiving inductive coil was within 10 cm of the active transmission coil placed beneath the home cage. Passive power draw of the system during operation ranged from 100 to 200 µW; however, during active protocol the power draw depended greatly on stimulation parameters, such as duty cycle, stimulation amplitude, and pulse width, with higher values increasing power requirements. One pilot system was assembled and subsequently leak tested while submerged in saline for a period of 48 h. The device functioned successfully throughout the test phase and disassembly revealed no obvious water penetration to the electronic housing. We also developed ultra-low bending stiffness electrode arrays with gold conductive traces via soft electronics fabrication techniques (Fig. 1). Arrays were connectorized via cold welding to an anisotropic conductive film (ACF; Elform Heat Seal Connectors) ribbon cable. The array lead was designed to be less than 1 mm wide in accordance with the width of the rat spinal cord and contained 8 electrode contacts (200 µm diameter each) that were spaced 500 µm apart in a 4 × 2 grid (Fig. 1, Supplementary Fig S1). Addition of more components and re-design of the printed circuit board (PCB) necessitated a second polyether ether ketone (PEEK) case for housing the battery and inductive charging electronics. The microcontroller and microstimulation system were independently housed from the battery and inductive charging circuitry each in a 2 cm^3^ case connected via 2 encapsulated braided stainless wires.

Telemetry and control of multiple devices was encoded over a 2.4 GHz digitally modulated radio transmission via a single USB communication module. Every minute, a radiofrequency burst from the stimulation module returned information on protocol status, impedance, temperature, and battery. Operation of the device and protocol encoding was performed via a custom user interface for ease of use. All internal electronic components were pre-assembled, gas sterilized with Ethylene Oxide, and then sealed with ultraviolet (UV) curable epoxy. Following assembly, each device was immersion tested in room temperature phosphate buffered saline for leak testing and subsequently surface sterilized for implantation.

### Surgical implantation of stimulation arrays

Two surgical approaches were considered for implantation of the designed electrode array, a direct cervical approach and an indirect thoracic approach where the array was threaded along the ventral epidural surface to the cervical region. The direct cervical approach presented issues given that cervical roots are more closely positioned at the cervical level than the thoracic or lumbar regions. The close proximity of the cervical roots increased the risk of unacceptable neural injury when placing a 1 mm wide flexible array. Further, epidural venous bleeding encountered with a wide laminotomy was difficult to control and, together with space occupying hemostatic agents, obscured visualization of lead insertion. Therefore, we discarded direct surgical placement of an electrode in the cervical region in favor of an indirect thoracic insertion.

**Figure 2:**
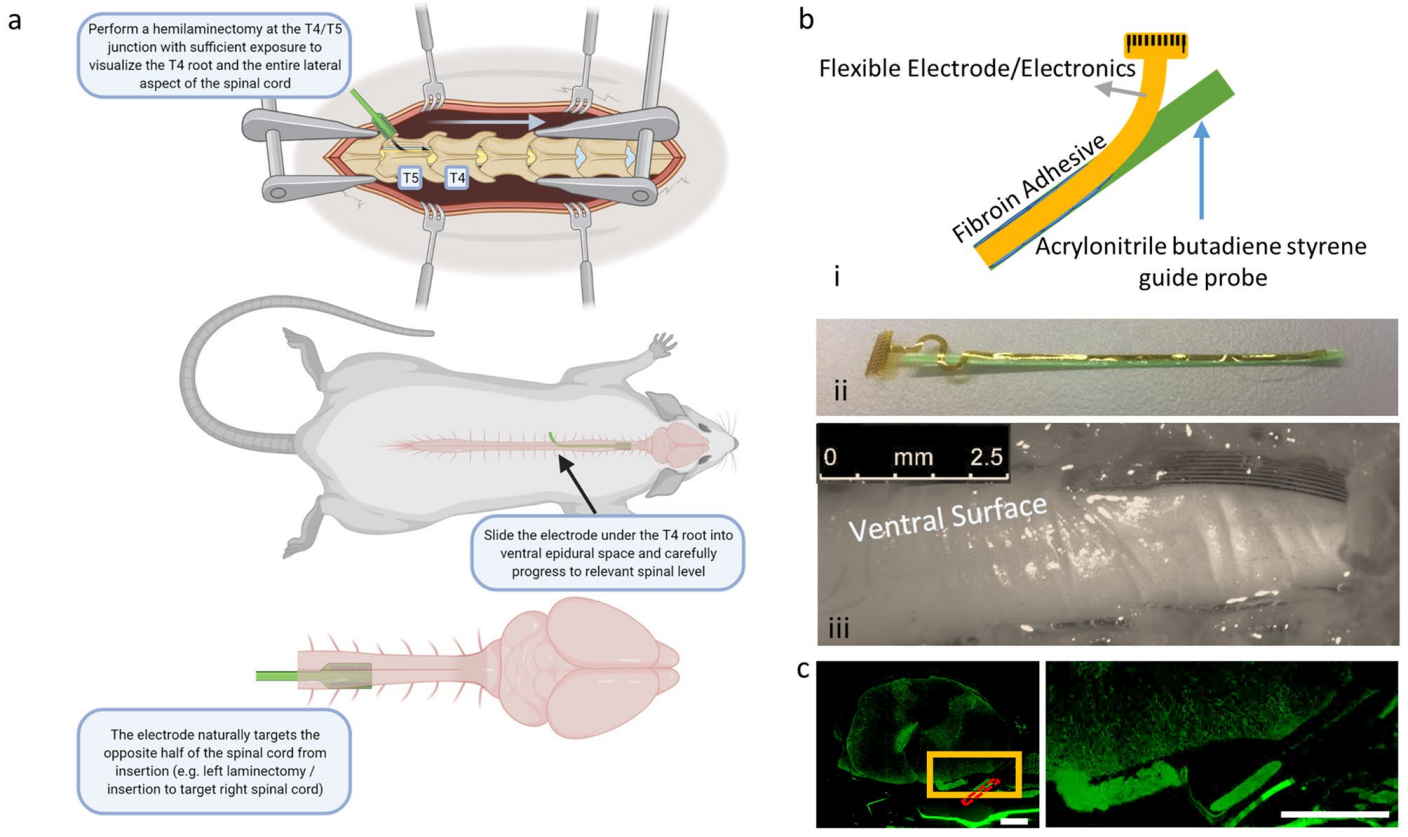
Surgical approach. (**a**) We developed a technique to insert highly flexible electronics into the ventral aspect of the cervical rat spinal cord using a dorsal thoracic approach. (**b**) (i) low bending stiffness biocompatible arrays were fixed to a 3D printed guide probe with biocompatible silk polymer fibroin to (ii) aid in insertion. (iii) Following 2 months of implantation, the array remained positioned at the C5/C6 spinal level lateral to midline. (**c**) IHC stains for GFAP revealed low bending stiffness arrays do not exhibit enhanced GFAP signal (green) near the spinal surface most proximal to the array, though arrays do appear to become encapsulated in connective tissue over time. Red dashed box—approximate array location, yellow box—location of inset, *all scale bars indicate 500 µm.

The indirect approach involved a more caudal thoracic T4/T5 hemilaminotomy, exposing the lateral thecal sac and spinal canal, allowing the array to be threaded laterally into the ventral epidural space and advanced as far as the C4 level without significant obstruction (Fig. 2). At the T4/T5 level, there was sufficient space between spinal roots to allow insertion of a 1 mm array between the T4 and T5 roots without damage or irritation to either. A low bending stiffness electrode array was used to prevent local injury by more closely matching the elastic modulus of the spinal cord. Increased flexibility made insertion as a stand-alone device impossible. Therefore, arrays were attached to a higher bending stiffness guide probe with sterile 5% fibroin silk polymer in order to be inserted into the spinal canal as a unit. The polymer allowed for transient attachment of the array to a substrate; however, the polymer naturally degraded over several minutes in body temperature saline. We tested different adhesion protocols in order to determine the best method for bonding the soft electronics to the guide probe and found that increasing amounts of fibroin solution not only lengthened crosslinking times, but also contributed to stability of the bonded system to physical perturbation. We further found that higher temperature crosslinking increased time to degradation; however, higher temperatures resulted in less physically stable bonding of the electronics to the probe (Supplementary Fig S2). The stabilized probe and array were positioned ventrally and directed cranially at a 45° angle with respect to the long axis of the thecal sac/spinal canal between the T4 and T5 roots and threaded circumferentially in a rostral direction until a fiducial was visualized at the laminotomy surface, indicating placement reached 2 cm rostral of the T4/T5 insertion site (Fig. 2). The joined probe and electrode array were threaded around the spinal cord from the dorsal approach between the T4 and T5 roots and localized in the ventral epidural space. Once in the epidural space, the probe could be freely mobilized rostrally up to the high cervical level. While spinal ligaments in the ventral space may present some anatomical barrier, we were able to reliably position the probe in the upper cervical level with readjustment and careful retraction and advancement. Given the consistent lateralization of the probe to the opposite aspect of the spinal cord from the side of insertion, it may be that spinal ligaments or the Long-Evans strain of rat’s natural anatomy provide anatomical guidance in the ventral space. The thoracic insertion approach from the dorsal aspect threading the electrode ventrally to the stimulation site was used to generate all data presented in the manuscript. The described surgical technique was used to successfully place probes in the ventral space of the 26 rats used in this study (Supplementary Table S1).

### Acute and chronic testing of VSS stimulators

We performed acute physiological studies to examine the muscle activation using VSS as well as a proof-of-concept chronic study to demonstrate the feasibility of our surgical approach and array. In acute studies, where animals were immediately sacrificed following data collection, incremental marks were placed on the array lead using a permanent marker to visualize the rostral location of the electrode array relative to the T4/T5 spinal level insertion site. However, in the chronic pilot study, where animals recovered and were monitored over time, arrays were inserted from the T4/T5 spinal level to a fixed location at the C4/C5 spinal level, as determined by a bead of polydimethylsiloxane (PDMS) on the array lead that served as both a fiducial and a soft tissue anchor for the lead, allowing for reliable placement of the array at the ventral epidural space of cervical level 4/5 (Fig. 2). Using the T4/T5 insertion method, we were able to localize the array more rostrally or caudally depending upon the spinal level to be stimulated. For chronic implantation, a PDMS bead was positioned at the exposed lead and secured by suture to the lateral paraspinal muscular ligaments at T4. A strain-relieving loop of lead was threaded in a sub-cutaneous pocket on the haunch before connection to the electronic module. The geometry of the array lead resulted in a paracentral placement biased towards the contralateral side. Specifically, the anatomical geometry of the insertion angle and the structure of the spinal column guided the array lead to the contralateral side of the ventral spinal canal on which it was inserted. Therefore, targeting the muscles of the right forelimb was achieved via a left sided T4/T5 hemilaminotomy and left sided insertion of the probe. Arrays were reliably placed at the level of interest, then encouraged to detach via application of warm saline between the probe and array lead. An array was implanted, and a rat was monitored over a 2-month period in a pilot trial. The rat exhibited no altered gait or obvious discomfort during this period, and histological analysis revealed no obvious gliosis by glial fibrillary acidic protein (GFAP) staining at the array interface (Fig. 2). However, the array does become encapsulated over the life of the implant as evidenced by laminin positive structures forming in a pocket around the array (Supplementary Fig S3). The addition of inductive charging allowed for effective power transfer to the device in a home cage (Supplementary Video S1).

### Electromyography (EMG) analysis and mapping

**Figure 3:**
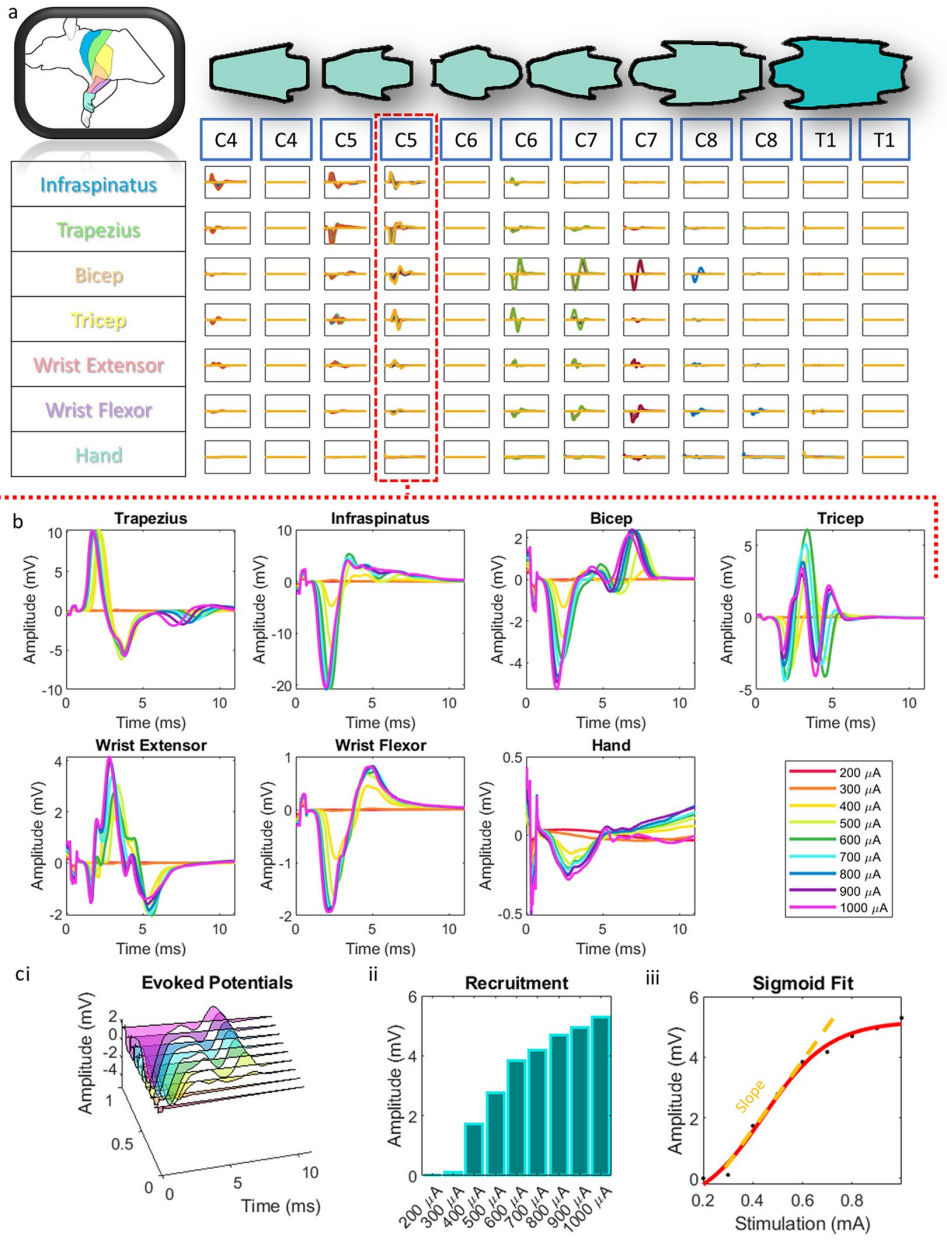
Point-to-reference EMG. (**a**) Grid of evoked potentials from each muscle at each stimulated position along the spinal cord using point-to-reference stimulation. Each square is an overlap of all of the traces at each stimulation amplitude. Each overlapped trace is the average of 20 repeats. *all graphs in grid are from 0 to 12 ms on the x axis and − 18 to 18 mV on the y axis. (**b**) An expanded view of (**a**) for a single stimulation point at the C5 level. (**c**) (i) For each muscle at each stimulation position, we took the recruitment curves at increasing stimulation intensities (ii) and extracted the evoked response amplitude. (iii) Then, we used a sigmoid fit function to extract the slope of the resulting dose response curve.

**Figure 4:**
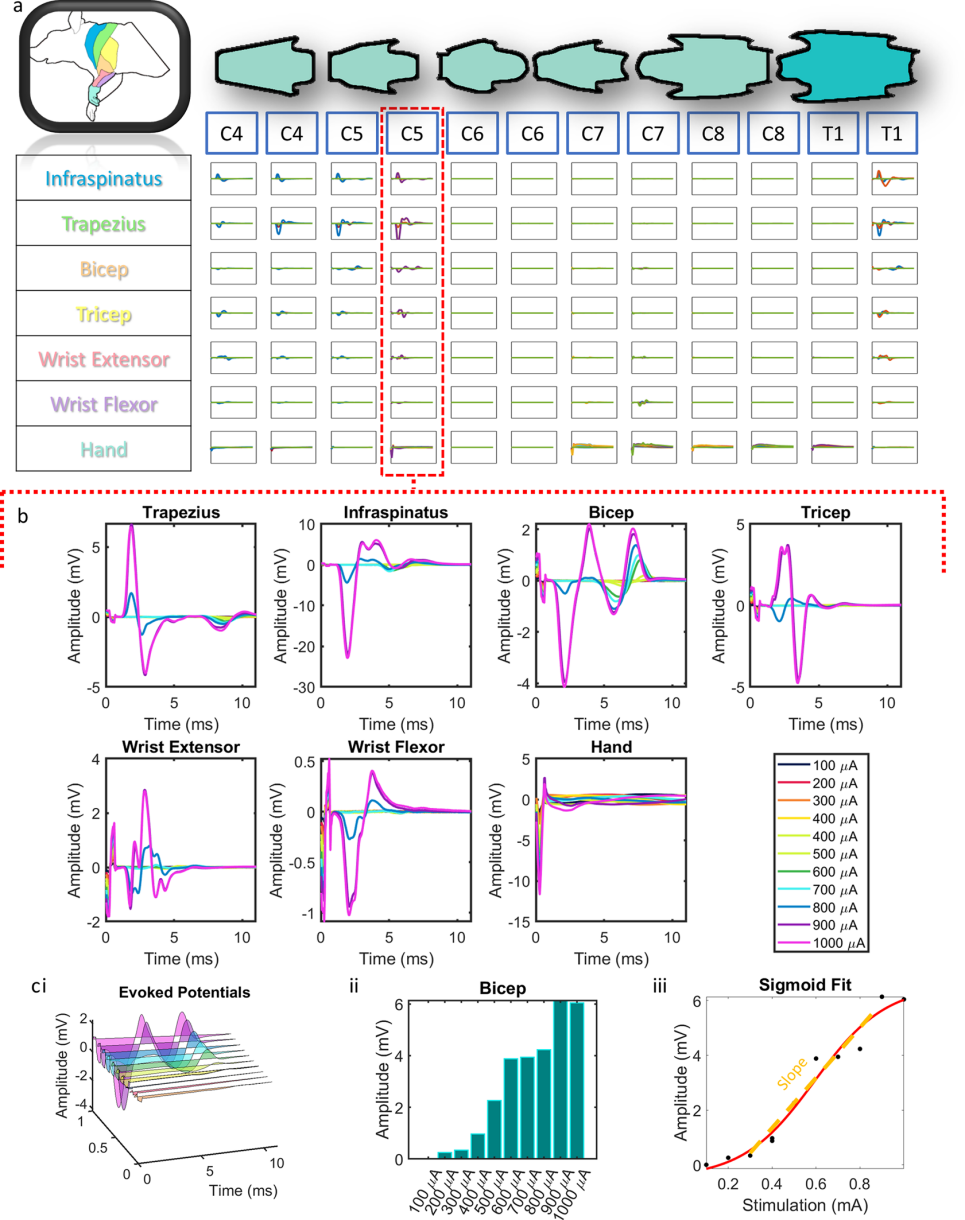
Point-to-point EMG. (**a**) Grid of evoked potentials from each muscle at each stimulated position along the spinal cord using point-to-point stimulation. Each square is an overlap of all of the traces at each stimulation amplitude. Each overlapped trace is the average of 20 repeats. *all graphs in grid are from 0 to 12 ms on the x axis and − 18 to 18 mV on the y axis. (**b**) An expanded view of (**a**) for a single stimulation point at the C5 level. (**c**) (i) For each muscle at each stimulation position, we took the recruitment curves at increasing stimulation intensities (ii) and extracted the evoked response amplitude. (iii) Then, we used a sigmoid fit function to extract the slope of the resulting dose response curve.

**Figure 5:**
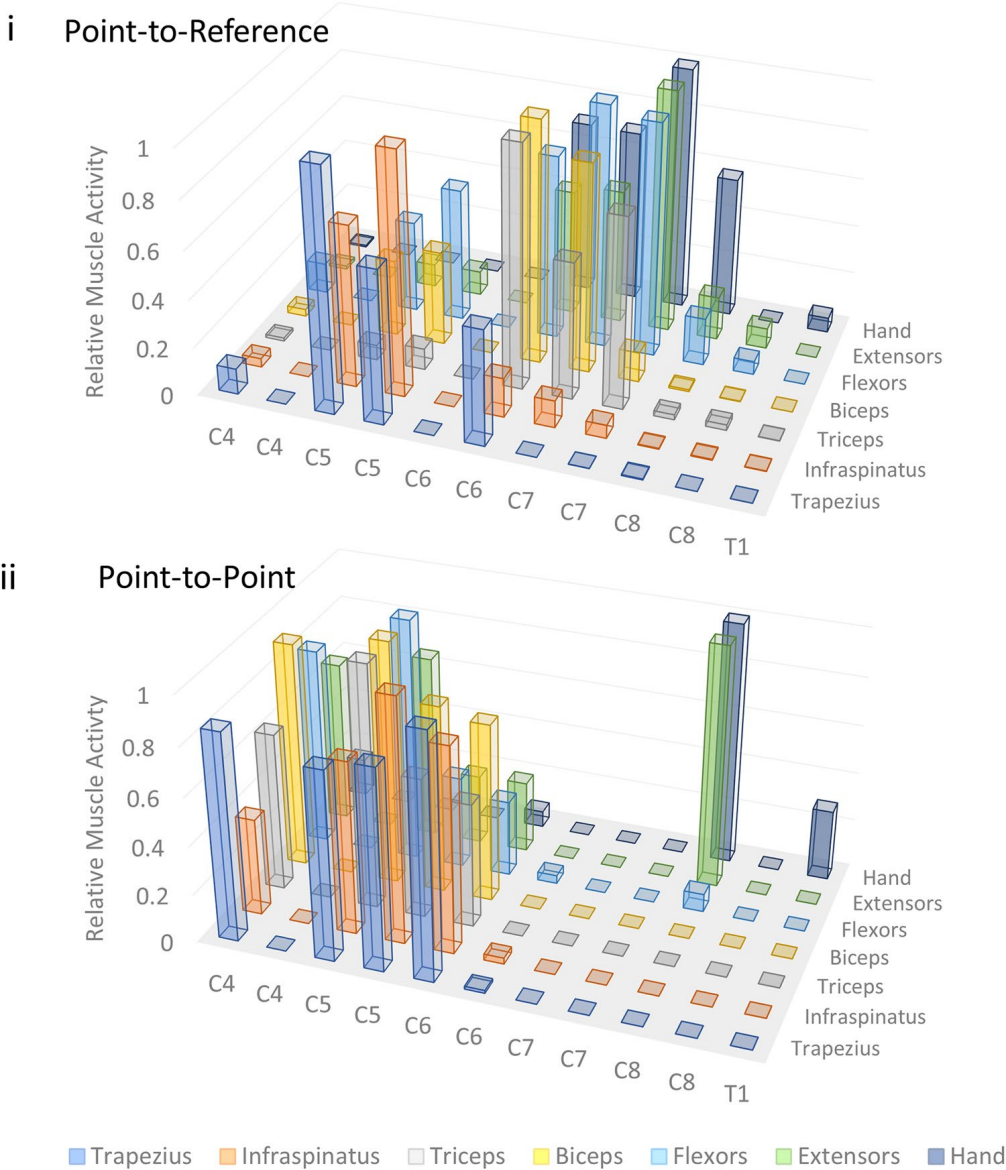
Point-to-reference and point-to-point mapping. The relative muscle activity was defined as the amplitude observed at a given level normalized to the maximal amplitude observed through an entire mapping session. The graph displays relative muscle activity at each spinal position using (i) point-to-reference and (ii) point-to-point stimulation modalities. *Measurements were collected at 1.5 mm increments so there are rostral and caudal measurements at each spinal segment except for T1 where there is a single rostral measurement. The difference in the distribution of activated muscles at each position reveals dramatically different responses at each spinal level using each technique.

Motor mapping experiments were conducted in order to determine the types of muscle activation that result from the rostro-caudal placement of our electrode array. Specifically, an acute preparation in a terminal experiment was used to mobilize and test array positions along multiple segments of the cervical and upper thoracic spinal cord. Evoked potentials were measured in the muscles of the forelimb by EMG to determine the motor activity at a given level of stimulation and segment position (Fig. 3). We stimulated at 1.5 mm intervals along the cervical and upper thoracic spinal cord to determine optimal spinal levels for targeting specific muscle groups and to demonstrate effective stimulation of the muscles of the rat forelimb. Mapping revealed effective activation of trapezius, infraspinatus, triceps, biceps, wrist flexors, wrist extensors, and hand muscles in the stimulated side of the spinal cord. In some cases, we achieved single digit activation when stimulating from the ventral surface (Supplementary Video S2). Recruitment curves demonstrated expected dose response patterns were generally sigmoidal in nature for both point-to-reference (Fig. 3) and point-to-point (Fig. 4) stimulation. A sigmoidal dose response curve was fit to the muscle recruitment responses for each muscle at each position to extract the slope of the curve fits. Figures 3C and 4C visualize an example of the workflow for the biceps muscle at the C5 level. At threshold, relatively few fibers associated with a muscle movement were engaged; however, as the current was increased, the EMG output indicated a representative increase in recruited spinal and motor circuitry associated with the measured muscle up to saturation where EMG output plateaued (Fig. 3). Thresholds ranged from 200 µA to 500 µA, with plateaus occurring by 1 mA in most cases (Fig. 3). Early component responses occurred within 2–4 ms of onset of stimulation with late components occurring after 4 ms, typical of observed latencies when stimulating the epidural cervical spine in monkeys^19^ and rats^36^. Proximal muscles (trapezius/infraspinatus) were preferentially activated at upper cervical levels; whereas, distal muscles (wrist extensors/wrist flexors/hand) were preferentially activated at caudal segments of the cervical and upper thoracic spine (Fig. 5).

### Multimodal stimulation

**Figure 6:**
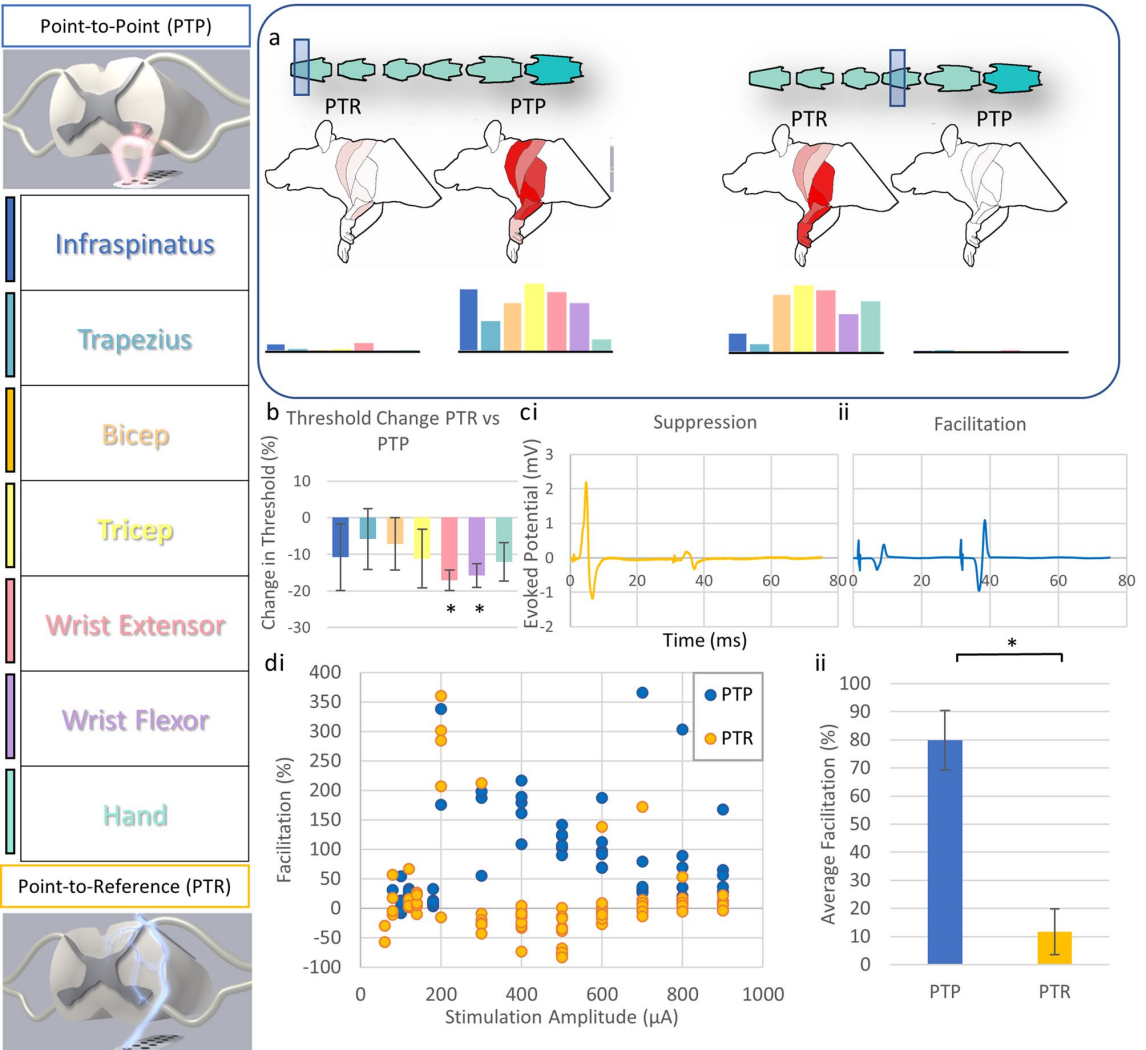
Modes of stimulation. (**a**) Examining the evoked muscle responses at each position along the spinal cord with both point-to-point (PTP) and point-to-reference (PTR) stimulation revealed positions where each mode induced distinct responses within the spinal cord. Each measurement demonstrates muscle activity at 400 µA constant current stimulation. The shaded box indicates the spinal level of stimulation and the intensity of red in the rat forelimb diagram indicates the level of activation of a given muscle group by relative muscle activity. This information is displayed below each diagram as a bar graph displaying muscle activity at each location normalized as a fraction from 0 to 1 where 1 is the maximal evoked response observed for each muscle at any stimulated position. (**b**) Thresholds were also affected by PTR vs PTP stimulation. For instance, at the C5/6 level, the thresholds of the wrist flexor and extensor were 15.7 and 17.1% lower respectively when stimulated at the same position in PTR vs PTP mode and there was a trend to lower overall thresholds in PTR mode. (**c**) Following a spinal stimulation doublet with a 30 ms interstimulation interval, we observed changes in the second evoked potential where the second amplitude was (i) lowered indicating signal suppression or (ii) raised indicating spinal facilitation. (**d**) (i) We analyzed the relative facilitation/suppression observed in both modes at various stimulation intensities and observed, (ii) PTP mode resulted in a greater degree of spinal facilitation.

**Figure 7:**
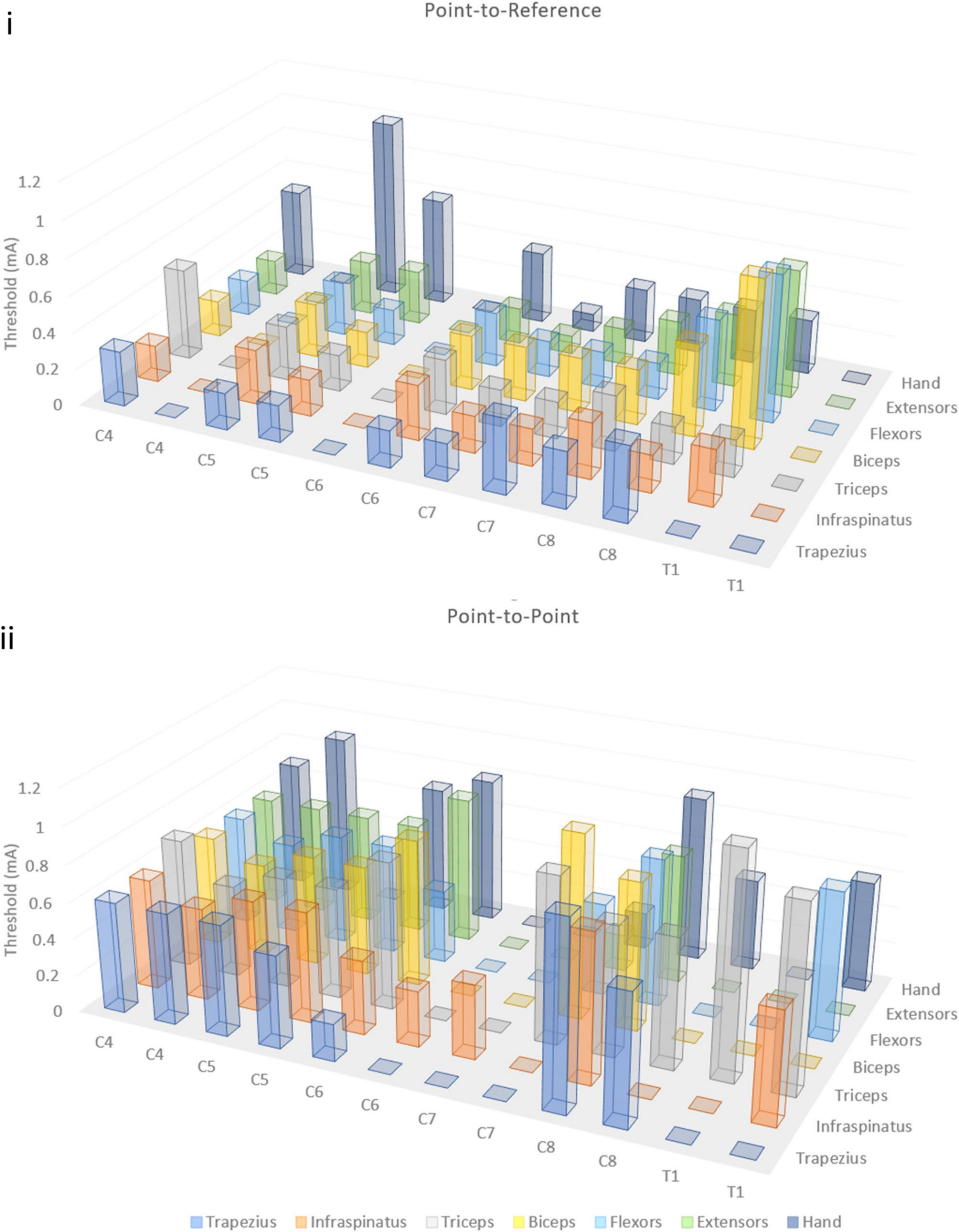
Point-to-reference and point-to-point thresholds. The threshold of a muscle was defined as the stimulation amplitude at which an evoked potential was observed. The graph displays thresholds for each muscle at each spinal position using point-to-reference (top) and point-to-point (bottom) stimulation modalities. *Measurements were collected at 1.5 mm increments.

We examined whether relative position of anode and cathode might play a role in the circuits targeted during spinal stimulation. Evoked potentials via spinal stimulation revealed two unique modes of delivering stimulus to the ventral rat spinal cord at a single position. When anode and cathode were proximally located on the ventral aspect of the spinal cord (point-to-point), evoked responses were substantially different than when the cathode was positioned further away (point-to-reference) (Fig. 6). Point-to-reference mapping revealed optimal activation of the muscles of the forelimb from the C5 to C8 spinal levels. Point-to-reference stimulation resulted in coincident activation of at least three muscles simultaneously at all observed levels, with the exception of the C8 level. Activation thresholds for forelimb muscles were lower for point-to-reference stimulation compared with point-to-point (Fig. 7), with the average threshold of activation of all forelimb muscles at each point tested being 314.5 ± 21.9 µA for point-to-reference and 639.3 ± 197.0 µA for point-to-point stimulation. Effective stimulation of a muscle was defined as muscles with thresholds under 500 μA at a given position, and we observed effective stimulation of more muscles at nearly every position using point-to-reference stimulation. Point-to-point stimulation revealed optimal activation from C4 to C6 levels and selective activation of the hand and wrist extensor muscles at the C8 level. Point-to-point, but not point-to-reference, allowed spinal level separation of hand and wrist versus upper limb and back musculature. Optimal activation was more rostral in point-to-point stimulation compared to point-to-reference activation (Fig. 6, Supplementary Video S3). During point-to-reference stimulation, there was a general activation of multiple muscle groups across nearly the entire length of the stimulated spinal cord; however, stimulation provided more selective activation of muscle groups at the C4/C5 levels. During point-to-reference stimulation, general activation occurred rostrally, with a sharp and selective activation of the musculature of the wrist and hand when stimulating at the C7/C8 levels. There was little activation of the muscles of the forelimb at the C6 level when using point-to-point stimulation; however, point-to-reference stimulation initiated some level of activation of all muscles at this level. We compared relative changes in thresholds when the electrode was placed at the C4/C5 level where all measured muscles could be activated at a single position in point-to-point and point-to-reference modes. We found a trend of reduced threshold when stimulating in point-to-reference mode compared with point-to-point, with significant reductions in threshold for the wrist flexor (− 17.1 ± 2.8%, p = 0.026, n = 3) and wrist extensor (− 15.7 ± 3.2%, p = 0.040, n = 3) muscles (Fig. 6). Spinal doublets delivered with 30 ms intervals revealed a change in the facilitation/suppression patterns of the evoked potentials when delivered in point-to-point versus point-to-reference mode. We compared the relative facilitation (percent increase) of the second evoked response with respect to the first response in both point-to-reference and point-to-point modes at varying stimulation intensities and discovered that point-to-point mode induced a greater degree of facilitation compared with point-to-reference mode (79.8 ± 10.6% vs 11.6 ± 8.1%, p < 0.001, n = 3).

## Discussion

The VSS system represents a new tool for stimulating the ventral surface of the spinal cord. Given its versatility and compatibility in the awake, behaving rat, this stimulation system is likely to add considerably to our understanding of motor circuity physiology in the healthy and injured nervous system. It is a completely wireless system capable of being fully implanted in a freely mobile rodent and of supplying stimulus to the ventral surface of the spinal cord. Epidural DSS strategies necessarily recruit propriospinal and sensory fibers and demonstrate less general specificity when compared with subdural and intraspinal stimulation approaches^19^. Intraspinal approaches have been shown to activate 1a afferents as well, and they likely operate through trans-synaptic mechanisms to activate motor neurons^37,38^. Still, the relative proximity of ISMS leads to functional circuits is the most likely reason for the relative increase in motor specificity when compared with DSS. At the very least, DSS approaches currently lack specific targeting mechanisms for discrete activation of motor pools, limiting effective targeted rehabilitation strategies^19,39^. ISMS wires offer an opportunity to improve targeting of individual muscle groups; and, in our group’s previously published work, we have demonstrated that neuromodulation by ISMS wires can produce sustained improvement in voluntary upper limb reaching and grasping even after termination of therapeutic stimulation^14^.

Nevertheless, parenchymal implantation of electrode wires requires invasive surgery and carries the risk of infection, tissue damage, and scarring that could worsen over time. Here, we developed a completely wireless, ventral spinal approach to allow for more flexible targeting of motor associated spinal circuitry, while maintaining safety and translational potential of an epidural approach. We posit our VSS stimulator can operate in two modes: (1) point-to-point, a more selective stimulation strategy that likely targets motor neurons and their inputs directly and (2) point-to-reference, which likely produces a more general activation that is similar to proposed DSS mechanisms. Upper limb EMG measures indicated that a multichannel thin film electrode array placed in the ventral epidural space allowed for diverse stimulation strategies of the ventral spinal surface. We have established an untethered and light weight device that is fully programmable and capable of stimulation studies including assessment of stimulation coincident with motor activity. A wireless system can be easily applied in behavioral assessments and physiologic experiments of freely moving animals. Altogether, in conception and execution, the VSS approach provides many benefits for research into potential therapeutic spinal stimulation.

A low-bending stiffness electrode array was well tolerated for over 2 months in a single animal, which is proof of concept that a more flexible material can be made to safely interface with the ventral surface. Given the low bending stiffness of the array lead, we needed to develop a means for insertion of a floppy material for blind axial insertion along the ventral surface. The low bending stiffness array we fabricated lacked the physical stiffness to be axially guided along the surface without additional support. Using a silk fibroin adhesive, we were able to attach the flexible device to a more rigid probe for blind insertion. The concept of using silk fibroin as a polymer for fabrication of flexible electronics systems to interface with the CNS is not new^40,41^, and as an adhesive it has been used to facilitate physical insertion of flexible electronics into the brain^42^. We adapted such techniques to curve the electronics around the lateral aspect of the spinal cord into the ventral epidural space and stably place the soft electronics to the desired site of stimulation. Using this technique, we observed no obvious behavioral complications over a 2-month period. Histological assessment revealed limited gliosis by GFAP staining in the area adjacent to the electrode array, however we did observe encapsulation of the array after 6 weeks of implantation (Supplementary Fig S3). The approach outlined here was effective at placing soft electronics in proximity to the rat spinal cord without damaging the electronics or the spinal cord. Such an approach might be adopted for interface of other fragile systems to challenging access points.

Our approach may also have clinical utility for placement of soft, flexible electronics in proximity to sensitive tissues in human patients. The primary reason we considered a dorsal to ventral surgical approach in our studies was the surgical challenge and increased potential for complications during an anterior exposure of the cervical vertebral foraminal space in a small rodent. In addition, due to the close proximity of dorsal roots in the cervical region of the rat spinal cord, surgical retraction of sensory roots is a necessary risk to accommodate the insertion of an electrode array circumferentially from the dorsal to the ventral vertebral foraminal space. Increased spacing of roots in the thoracic region gives more room to safely guide a flexible probe around to the ventral surface before mobilizing the array rostral. Innovation in human neurosurgical practice has made it feasible to easily access the ventral aspect of the spinal column and cervical spinal cord when the patient is placed in the supine position^43^. Therefore, a dorsal to ventral surgical approach may not be necessary for placement of devices or electronics within the anterior aspect of the vertebral foramen in humans. Still, the spacing between human cervical dorsal roots is much larger than the rodent, thus increasing the feasibility of a dorsal to ventral or even a circumferential insertion of an electrode in humans.

Examining the evoked potentials in acute preparations in rats revealed the functional capabilities of an epidural VSS. Mapping observations aligned with known distribution of motor pools in the rat cervical spinal cord and were in line with mapping studies, demonstrating the distribution of motor activity in the cervical spinal cord elicited by ISMS^4^. These data indicate that the geometric location of the VSS primarily elicits activity from local circuitry and not through distant circuitry via fluid or other means. Therefore, VSS may be used to target specific motor associated circuitry for conditioning with limited off target effects compared to less invasive, but more general, stimulation strategies. Further, while selectivity of spinal circuitry is generally lower from the ventral epidural surface compared to ISMS, it is important to note that ventral neuromodulation across the dural surface allows significant versatility to target select motor movements of the upper arm and hand. In addition, we saw less ectopic stimulation of muscles when using point-to-point mode; whereas, point-to-reference mode most often yielded fused contractions, indicating less specificity and potentially a more general activation of spinal circuitry.

We observed a critical stimulation behavior for our VSS systems where cathode proximity and placement resulted in altered activation of spinal circuitry, indicating that it may be capable of two modes of operation in the ventral space at a single spinal position. Given the importance of regaining hand and wrist function following cervical injury, a means to target spinal circuitry associated with hand and wrist muscles is critically important^14,15,17^. Location of the anode and cathode are crucially important in spinal stimulation and the positioning of each can greatly affect the resulting current field and neuronal response^44^. Still, very few studies have examined stimulation in the ventral epidural space since such stimulation typically requires higher levels of current^19^. By manipulating the locations of the anode and cathode relative to the ventral spinal surface, we discovered two distinct modes of operation: point-to-point and point-to-reference stimulation. Stimulation in point-to-point mode yielded discrete and pronounced activation of the wrist extensor and digit muscles compared to point-to-reference stimulation at the C8 spinal level. Importantly, at the same spinal level, stimulation in point-to-reference mode induced strong activation of digit, wrist flexors, wrist extensors, and tricep muscles. This activity suggests a multimodal functionality of our stimulator. Further, the evoked responses seem to indicate distinct spinal circuitry is involved with each different mode of stimulation. At several levels, particularly in point-to-point mode, there appear to be zones with little to no evoked responses in the muscles of the forelimb (Fig. 7). It is possible that with point-to-point stimulation, the current distribution is more focused. As such, it may be that motor pools are less likely to be activated by the more concentrated current. The trade-off is a higher degree of specificity with the possible cost of limited or insufficient activation of targeted circuitry. It may also be that spinal anatomy at levels where no response was observed, particularly around C7, precluded effective stimulation in point-to-point configuration. We also observed stimulation regions (e.g., C4 and C6) where the point-to-point configuration more effectively stimulated forelimb muscles in comparison with point-to-reference mode (Fig. 7). It is likely that the current density distribution within the spinal cord is fundamentally different in each mode, resulting in effective stimulation of different regions of the spinal cord. Future work needs to be done to directly compare mapping from the dorsal surface with that of the ventral map created here. Still, these data show the utility of VSS to interrogate the location of spinal circuitry, to determine the latencies for muscle activation, and even to directly activate discrete hand muscles. VSS-generated maps need to be further explored in the awake, behaving animal and in the context of development or neural injury in order to fully understand the relationship between activated circuitry and position of the anode and cathode relative to the ventral spinal surface.

While a direct comparison of thresholds may not be an accurate comparison between studies, given that current distribution depends on the pole-to-pole placement (relative positions of anode and cathode), the size of the poles, and the species and distribution of stimulated tissues, our results are still relatively in line with published observations of cervical stimulation strategies in monkeys^19^. Point-to-reference stimulation yielded largely fused contractions at lower amplitudes, meaning that a more general activation of spinal circuitry occurred. This observation is consistent with thresholds evoked from the dorsal surface by Jackson and Sharpe in 2014^19^. Whereas, point-to-point stimulation required significantly higher current to initiate contraction with more separation between thresholds of muscles of the hand and wrist (Fig. 7, Supplementary Table S2). As such, selective targeting evidenced in point-to-point mode may be through direct activation of α motor neurons and their synaptic inputs. This observation is consistent with the findings of Jackson and Sharpe who posit that ventral epidural stimulation acts through a different circuitry than DSS. They further observe that this difference in activation is not likely due to dural anatomy differences, but rather a difference between proximal circuitry. It is possible that distributed current summates in lower threshold axons and neural cell bodies, thus producing a more general activation of the spinal cord. This hypothesis is bolstered by our observation that distal placement of a reference wire generated more general contractility at lower thresholds. When anode and cathode are proximally located on the ventral aspect, we observed much higher thresholds of activation (Fig. 7, Supplementary Table S2) and more selective targeting of muscles, particularly of the wrist flexor and hand muscles. Point-to-reference resembles monopolar stimulation given the distal location of the reference wire. The observations of lower thresholds and lower specificity are consistent with clinical findings that bipolar cortical stimulation required higher current to initiate muscle contractions when compared with monopolar stimulation at the same site^45,46^. Further, a study of dorsal root stimulation in the spine presents evidence that bipolar stimulation may offer a higher degree of spatial selectivity compared with monopolar stimulation^47^. The study also observed that polarity of stimulation caused differential responses, indicating that electrode position along the spinal cord is critical for modulating recruited circuitry with different spinal stimulation modalities. Importantly, we hypothesize that different circuitry is activated when stimulating in point-to-point mode relative to point-to-reference mode, similar to previous observations by Jackson and Sharpe comparing ventral and dorsal stimulation of the cervical spine in monkeys^19^. It is possible that point-to-reference mode operates similarly to epidural dorsal stimulation, which is thought to primarily initiate polysynaptic sensorimotor activation through dorsal spinal afferents in humans^48^. The observation that spinal doublets in point-to-point mode exhibited higher degrees of evoked response facilitation compared with point-to-reference mode (Fig. 6) is consistent with a higher contribution of monosynaptic and direct motor neuron activation following ventral spinal stimulation in the Jackson and Sharpe study. We concede that more work is needed to prove this hypothesis; however, if point-to-point and point-to-reference stimulation act independently on unique circuitry, there is utility in combined stimulation and control strategies leveraging both general (point-to-reference) and selective (point-to-point) stimulation.

Indeed, several spinal stimulation control strategies have been employed to leverage unique types of stimulation and to adaptively modulate spinal activity, thereby improving function after SCI^49–53^. Neuromodulatory approaches are hypothesized to generally lower the threshold of activation, allowing for supraspinal control of patterned functional circuits, particularly during standing and walking^17,54,55^. Adaptive modulation of these circuits may provide enhanced benefit for rehabilitation. The addition of feedback and a control algorithm could possibly combine general (point-to-reference) stimulation with direct motor neuron activation to strengthen circuits in a task dependent manner.

More work is needed to address the circuits involved using the different approaches and, indeed, we have not yet performed a direct comparison of activated circuitry using VSS in the uninjured and injured spinal cord. A direct comparison of point-to-reference vs DSS at the same spinal level may go a long way to elucidate which circuits are involved. Certainly, a follow-up study that directly compares different stimulation approaches utilizing stimulation trains and doublets, neurotransmitter agonists, and SCI could provide much detail on the types of circuits activated by each stimulation route. Regardless, the approach that we present here, which may differentially target two distinct motor associated circuitries in the spinal cord, could enable combined general and selective activation with a single lead placement, allowing for more complex multifaceted therapeutic stimulation strategies.

Given the unique and multifunctional nature of our system, we believe it to be a potentially powerful tool for stimulation of the rat cervical spinal cord, particularly after SCI. Being completely wireless, functionally extensible, designed for long operation, and capable of delivering complex and continuous stimuli to the ventral aspect of the spinal cord, we believe the system will have broad appeal to neurophysiologists interested in the physiology of motor segment development, activity-dependent plasticity, and the potential for mapping the pathophysiology of SCI. To date, this is the first reported wireless system designed to target the ventral aspect of the spinal cord with epidural stimuli. Further, the system can be modified to include intraspinal stimulation channels, and our newest design includes a high current line for optogenetic studies.

## Conclusion

Neuromodulation to test and restore function of the upper limb is just beginning to be explored in the clinic and in rodent models. The VSS system designed here is likely to be not only an important tool in and of itself, but also a complimentary system to support neurorehabilitation, secondary stimulators, brain-computer interfaces, or exoskeleton approaches. Our data indicate that VSS is a safe and functional tool for targeted stimulation of specific motor units of the forelimb, especially the paw and wrist, which may not be achievable with a DSS approach. The optimized surgical approach, wireless stimulator versatility, and chronic implantation potential of the VSS system will make it an accessible system to research labs. In the near future, rigorous testing of the therapeutic potential of VSS is warranted in models of SCI, head injury, and stroke.

## Materials and methods

All procedures were approved by and conducted in accordance with the policies of the Institutional Animal Care and Use Committee (IACUC) at the Houston Methodist Research Institute (AUP-0119-0006) and reported in accordance with Animal Research: Reporting of In Vivo Experiments (ARRIVE) guidelines.

### Stimulator design and assembly

A wireless stimulation module was designed to deliver a stimulus to the spinal cord. The module consisted of an ultra-low power MSP430 microcontroller (Texas Instruments, TX) with an embedded control algorithm for the generation of a custom square wave stimulation sequence. A communication protocol designed by Indus (Indus, TX) was employed for low power operation. Communication occurred over the Industrial, Scientific, and Medical (ISM) band (2.4–2.5 GHz) with 2 Mbps data rate asymmetric communication. In order to preserve power, the onboard radio of the stimulation module was activated once per minute to update protocol instructions and return onboard temperature, power supply voltage, and operating status. The stimulator module was designed to provide 8 channels of independent stimulation with floating connections to allow selection of any channel as the anode and cathode with biphasic stimulation. Stimulation current was programmable from 0 to 5000 µA with a 13 V compliance. Biphasic stimulation timing was programmatically limited to 280–65,535 µS for the anode to cathode pulse and 60–65,535 µS for the interstimulus interval and cathode to anode pulse. The control module was linked to a power module with inductive wireless charging developed from technologies described by Ghovanloo et al.^35^ and was connected to a rechargeable lithium ion battery. The adapted scheme used a single active transmit coil on a custom PCB designed to be placed under a rodent home cage and a single receive coil operating on the 13.56 MHz band. Nearfield power transmission ranged based on relative position of the transmit and receive coil extending from 0 to 10 mW when the receive coil was positioned within 10 cm of the transmit coil. The stimulation module was housed in a PEEK enclosure. All internal electronic components were pre-assembled, gas sterilized with Ethylene Oxide, then sealed with medical grade UV curable epoxy (1180-M-T, Dymax, CT). Deformable electrode arrays were connectorized to the control system via an exposed trace silver nitrate ribbon cable cold-welded to both the wireless stimulation module and the custom array at 175 °C. Stimulation systems can be made available upon request and fabricated for purchase by Indus Electronics (Houston, TX).

### Fabrication of soft deformable electrode arrays

A glass substrate was mechanically washed with water, 3-indolepropionic acid (IPA), acetone, and O_2_ plasma (CS-1701, Nordson). The adhesion promoter (1 wt% (3-Aminopropyl)triethoxysilane (APTES, Sigma) aqueous solution) was spin coated on pre-treated glass at 3000 rpm for 30 s. Afterwards, polyimide (PI) (PI-2545, HD Microsystems) was spin coated at 1500 rpm on the glass and then cured at 250 °C for 1 h, followed by several applications of PI to achieve desired thickness. The Cr/Au (5/100 nm) were deposited by e-beam evaporator and then patterned by lithography and wet etching to define the geometries of the arrays. Next, a thin encapsulating PI layer was spin coated at 3000 rpm for 30 s. To pattern the encapsulating PI, the spin coated PI was first prebaked at 150 °C for 10 min, then a positive photoresist (AZ 5214, MicroChemicals) was formed on prebaked PI and selectively exposed (mask aligner) and developed (AZ 917 MIF MicroChemicals) for 1 min. During development, the unprotected PI was also dissolved in MIF 917. The top PI was fully cured after removing the photoresist by acetone. To pattern through the thick PI substrate, copper (100 nm) was used as a hard mask. After deposition and patterning of the copper mask, the sample was etched in O_2_ plasma for 1 h to isolate the arrays. The soft deformable arrays were finally released by buffered oxide etchant (BOE, 1:6, Transene) and washed with deionized water several times (Supplementary Fig S1). After drying, the arrays were interfaced with anisotropic conductive film (ACF, Elform Heat Seal Connectors) ribbon cables to a PCB, which was connected to an external amplifier for signal acquisition. To form a robust mechanical connection, the ACF was press cured at 170 °C for 1 min. The array was finally peeled off from the glass substrate carefully.

### Ventral epidural array insertion

All procedures were performed using female Long-Evans rats approximately of 4–5 months in age in accordance with guidelines provided by IACUC. The T4/T5 lamina were exposed and a partial lateralized laminotomy was performed on a caudal portion of T4 and rostral portion of T5 on the contra-lateral handedness side. Sufficient bone was removed such that the lateral surface of the spinal cord could be visualized. The array was inserted between the T4 and T5 roots with the electrodes facing the spinal surface. The T4/T5 level was selected for multiple reasons. Insertion below the T5 level resulted in significant bleeding due to obstructing vasculature. Above the T2 level, it was difficult to avoid root damage during insertion and stabilization of the array was more challenging. Following insertion at the T4/T5 level, it was possible to thread the array tip to any cervical level. The array was marked intermittently with a sterile marker to allow for both the measurement of the traversed insertion distance and the approximate placement of the array tip, which was then confirmed by intra-operative stimulation to induce visible movement or EMG responses. The array was looped and sutured to the dorsal spinal process at T5 to secure the device. The array housing container was placed in a dermal pocket and secured to the skin with sutures. The musculature was then closed, and a braided reference wire was threaded subdermally on the rostral axis and secured with sutures.

### Preparation of low bending stiffness electrode array probes

Using a LulzBot Taz 5 3D printer, guide probes were 3D printed with a thickness of approximately 60 µm, a width of 2 mm, and a length of 6 cm. Probes were designed to be stiff enough to allow axial feeding of the array along the ventral surface of the spinal cord without collapsing or bending into the spinal cord, yet flexible enough to allow the probe to curve around the lateral portion of the spinal cord and feed into the ventral epidural space. Acrylonitrile butadiene styrene (ABS) guide probes and soft arrays were gas sterilized with ethylene oxide (or ethidium bromide) prior to preparation for surgical insertion. To prepare the probes for implantation, 20 µL of 50 mg/mL silk fibroin filter sterilized solution in water (Advanced BioMatrix, CA) was applied to the ABS probe in a laminar flow hood. After placement, solution was air dried in a sterile petri dish for 15 min, then closed and sealed with parafilm.

### Insertion of low bending stiffness guide probe

Placement was confirmed through stimulation and evoked EMG potentials. Once in place, we separated the guide probe from the soft electronics while maintaining position and integrity of the components. Warm saline was applied gently to the exposed ABS/array, which allowed for separation of the two components over approximately 5 min. When separated, more saline was applied between the two components to facilitate further dissolution of the fibroin glue and to facilitate manual separation of the components. A second sterile 20 µm thick ABS separation element with the same length and width as the guide probe was inserted between the soft electronics and the original ABS guide probe to encourage parting of the two components both physically and by facilitating warm saline penetration between the two surfaces to enhance dissolution of the silk fibroin glue. Once the separation element was inserted to the full length of the guide probe, both were gently removed, leaving the array in place and tip location was re-confirmed using evoked EMG potentials.

### Electrophysiology recording and mapping

Recordings were performed on an air table inside a Faraday cage. EMG recordings were collected using a bipolar 32 channel electrophysiology system (Tucker Davis Technologies, FL). Arrays were directly connected to safe-touch connectors through a PZ5 NeuroDigitizer amplifier with a ± 500 mV signal input range. Stimulation signals were biphasic 300 µs pulses, with a 60 µs ISI where a cathodic pulse was followed by a charge balancing anodic pulse. Briefly, the animal was placed on a platform and the muscles of the stimulated limb were exposed by carefully teasing the skin off the muscles with Dumont forceps (Fine Science Tools, CA) and dissecting scissors. Needle electrodes were placed in the cervical trapezius, infraspinatus, tricep, bicep, wrist flexors, wrist extensors, and digit muscles of the stimulated forelimb. Samples were recorded at 50 kS/s through the PZ5 using AC differential coupling with a 0.4 Hz highpass and 22.5 kHz lowpass filter and a minimum of 20 evoked potentials were averaged for each data point per animal. Measured components of an evoked potential were detected and sorted through a custom MATLAB program (MathWorks, MA). Spinal stimulation arrays were connected to a uniaxial micromanipulator and the array was withdrawn in 2 mm increments in order to fully map the stimuli evoked at 0.5 mm increments along the spinal cord. Evoked latencies, amplitudes, and recruitment curves were collected for all 8 electrodes at each 2 mm interval along the spinal surface.

### Statistics and analysis

When analyzing thresholds, significant differences between raw data sets were performed using one-way analysis of variance with Tukey’s range test for post hoc analysis. Data are presented as means ± standard deviation of the number (n) of observations. Two-tailed significance was determined using a P value of < 0.05. When analyzing facilitation, raw data were processed using a non-parametric Wilcoxon-Mann–Whitney test to determine whether the distribution of relative facilitation differed between point-to-point and point-to-reference datasets. Two-tailed significance was determined using a P value of < 0.05. Sigmoidal curves were fit to recruitment datasets for each muscle at each stimulated position for both point-to-point and point-to-reference datasets using a custom MATLAB function^56^. All curve fittings and statistical analyses were performed using GraphPad Prism 7 (GraphPad Software, CA) and MATLAB (MathWorks, MA).

### Histology

Animals were pericardially perfused on a down draft perfusion table with heparinized phosphate buffered saline followed by 4% paraformaldehyde. Spinal cord tissues were extracted and post-fixed overnight, then cryoprotected using 10%, 20%, and 30% sucrose solutions. Spinal cords were embedded in Tissue-Tek OCT compound (Sakura, Holland) and snap frozen in liquid nitrogen. Samples were sectioned longitudinally with 20 µm thickness on a Cryostar NX50 cryostat (ThermoFisher, MA) and mounted on slides for staining. Slides were blocked with 10% Donkey serum (Millipore Sigma, MA) and 0.5% Triton X-100 (Millipore Sigma, MA) in phosphate buffered saline for 1 h at room temperature. Longitudinal 20 µm sections of spinal cord tissue were stained for rabbit GFAP (AB72650, Abcam, United Kingdom) at a concentration of 1:500 for 2 h, washed 3× in phosphate buffered saline for 7 min, and counterstained with donkey anti-rabbit AlexaFlour 488 (AB2313584, Jackson ImmunoResearch, PA) for 1 h. Sections were again rinsed 3× with phosphate buffered saline for 7 min each and coverslips were attached using 80uL of Fluoromount-G (Southern Biotech, AL). Slides were imaged using LAS X software suite on a Leica DMI8 inverted confocal microscope (Leica Microsystems, Germany).

## Supplementary Information

Supplementary Video 1.

Supplementary Video 2.

Supplementary Video 3.

Supplementary Information 1.

## Data Availability

The datasets generated in the reported studies and subsequent analysis and related scripts and materials are available from the corresponding authors upon reasonable request.
