# Supplementary material for: A wireless spinal stimulation system for ventral activation of the rat cervical spinal cord
Source: Sci Rep. 2021 Jul 21;11:14900. doi: 10.1038/s41598-021-94047-1 (PMC8295294; doi:10.1038/s41598-021-94047-1)
Supplement: Supplementary file 4 — Supplementary Information 1. [file 41598_2021_94047_MOESM4_ESM.pdf]

A Wireless Spinal Stimulation System for Ventral Activation of the Rat Cervical Spinal Cord: Supplementary Information

Hogan, Matthew K.*[1]; Barber, Sean M. [1]; Rao, Zhoulyu[1]; Kondiles, Bethany R.[1, 3]; Huang, Meng[1]; Steele, William J.[1]; Yu, Cunjiang[2]; Horner, Philip J.[1]

1. Department of Neurosurgery, Center for Neuroregeneration, Houston Methodist Research Institute

2. University of Houston

3. Department of Physiology and Biophysics, University of Washington

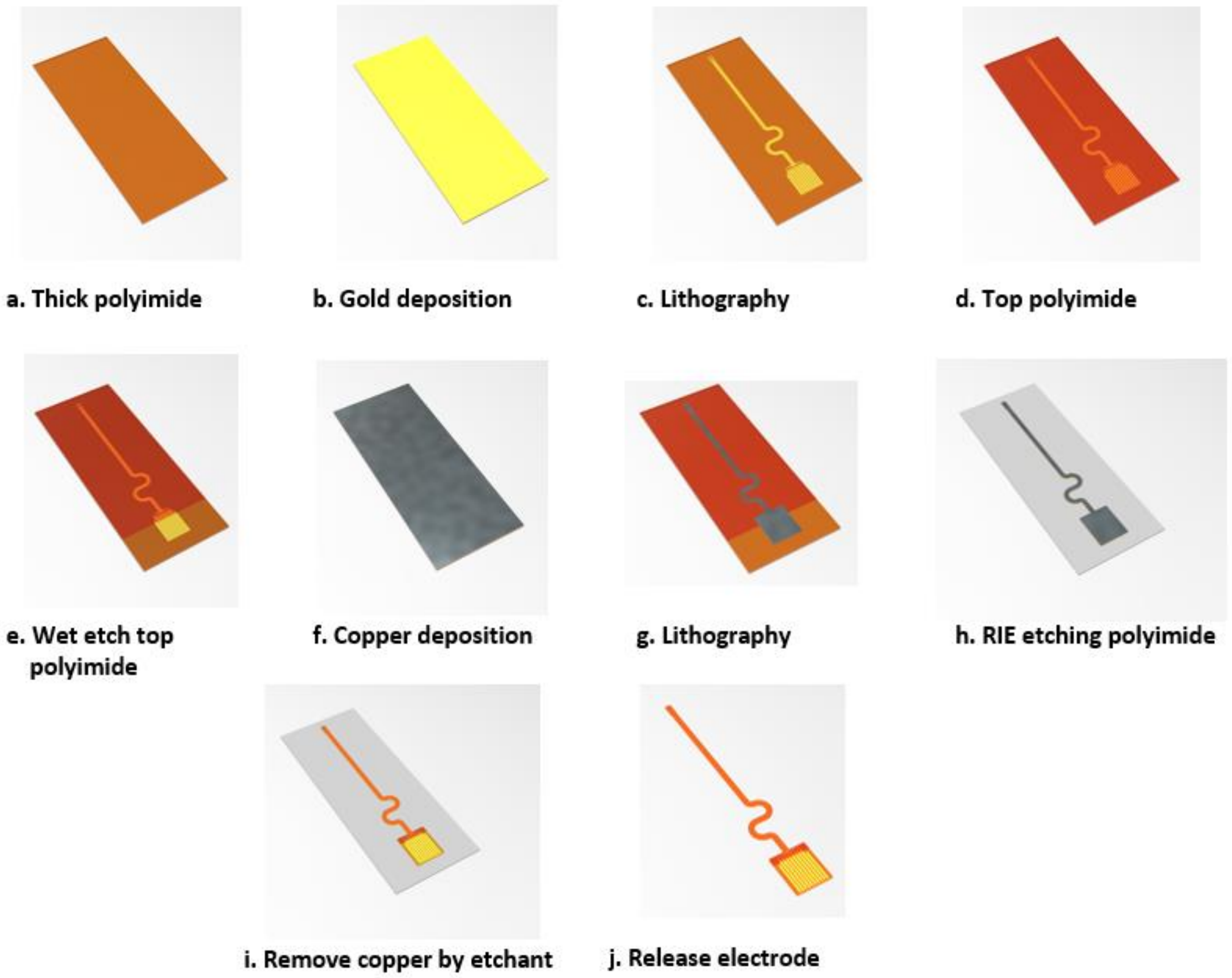

Supplementary Fig S1: Fabrication Steps for A Low Bending Stiffness Electrode. a) Spin coat an initial thick layer of polyimide as a substrate on a glass slide. b) Deposit a thin layer of gold by e-beam evaporation. c) Lithographically define and wet etch the electrode traces. d) Spin coat to apply a second thin film of polyimide. e) Wet etch to remove polyimide where gold is exposed. Next, f) deposit copper and g) lithographically pattern it to serve as a hard mask for h) O2 plasma etching to isolate the electrodes. Finally, i) remove the copper with chemical etchant and j) release the electrode from the glass substrate.

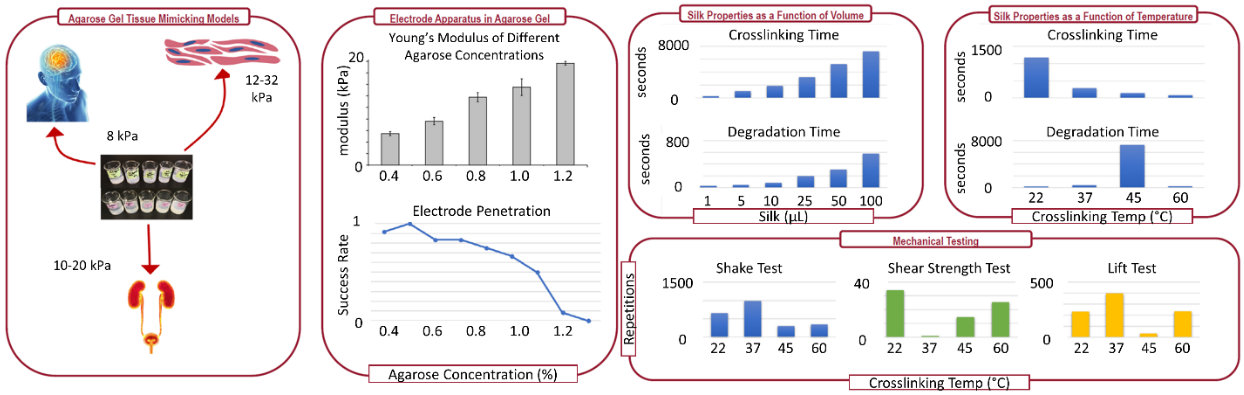

Supplementary Fig S2: Fibroin Adhesive for Soft Electronics Implantation. We examined an approach of using silk bonded to a 3D printed probe for placement of soft electronics in a variety of circumstances. a) The stiffness of agarose gel can be modified to match the Young's modulus of a variety of tissues. b) We tested the ability of our guide probe to penetrate various stiffness agarose gels while successfully deploying a soft electrode. c) Applying more silk to the probe required longer times to fix the electrode to the probe; however, once attached, larger quantities of silk were more stable and took longer to degrade in room temperature saline. d) We further examined how temperature would affect bonding time of 5 µL of silk polymer and found that increasing temperature increased the rate of bonding. Notably, at 45 °C the time to degrade in room temperature saline was increased. e) We examined the mechanical properties of probes crosslinked at various temperatures by shaking the probes (shake test), flexing the probes by bending (shear strength test), and lifting by the unattached portion of the electrode (lift test) until failure. Tests revealed that while crosslinking at 45 °C increases the time to degradation, the mechanical properties are not otherwise superior. As such, we opted for room temperature crosslinking as a balanced option for mechanical and degradation properties.

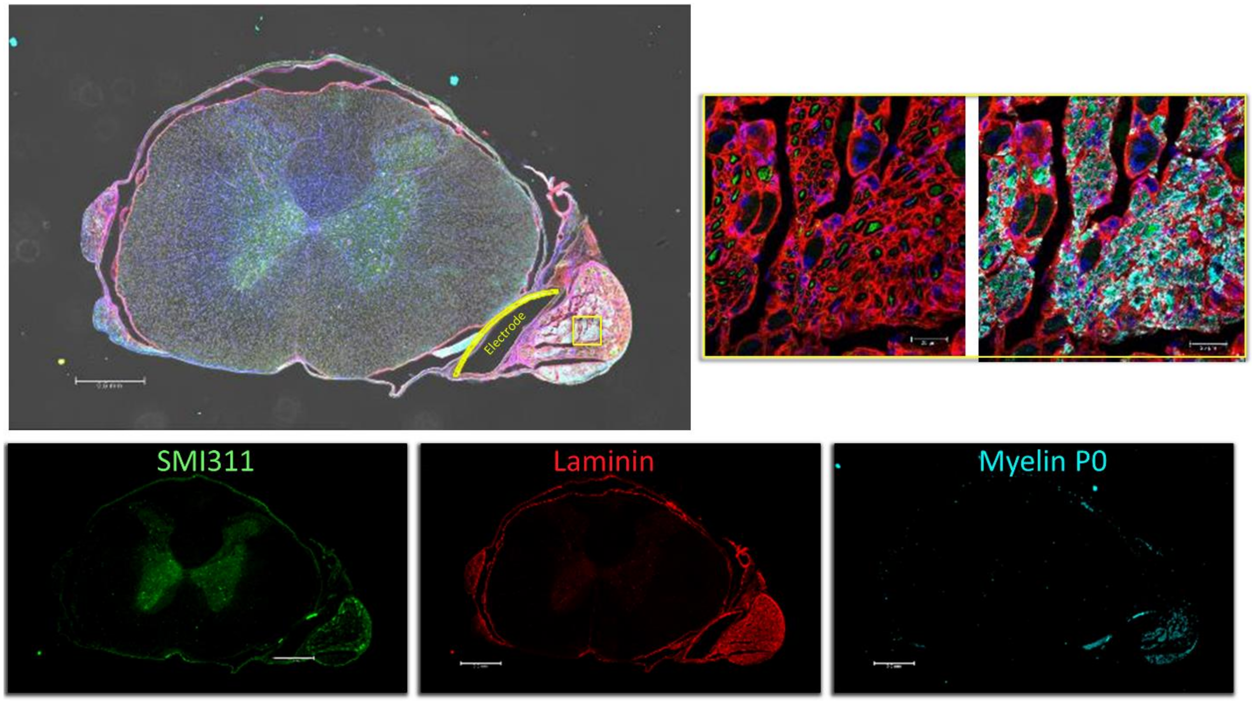

Supplementary Fig S3: Electrodes become encapsulated following prolonged implantation. Tilescan images of SMI311 (green), a neurofilament marker, laminin (red), a basement membrane marker, P0, a peripheral myelin marker (teal), and DAPI. The merged channels are shown in the top left and individual channels can be seen on the bottom; scale bars = 500 µm. Inset top left shows an enhanced view of a root structure with (right) and without (left) P0 label; scale bars = 25 µm. Sample collected 6 weeks post implantation. Laminin staining shows a basement membrane structure surrounding the electrode.

Implant Date	Number of Animals	Use	Notes
4-Nov-16	4	Pilot Surgery	We performed 4 surgeries to determine the appropriate surgical route while also achieving targetted stimulation of the muscles of only the left or right forelimb.
7-Apr-17	10	Effects of spinal stimulation on neural stem cell engraftment	Results were compromised. Animals in the stimulated group had neural stem cell grafts with greater fiber alignment along the rostro-caudal axis compared with unstimulated but given complications resulting from stiff electrodes in a mobilized free moving animal, we decided to discard the data.
3-Aug-17	1	Ephys Mapping	Data was captured using a different stimulation and recording system, new electrode configuration was subsequently designed so we decided to present the latter data.
16-Aug-17	1	Ephys Mapping	Data was captured using a different stimulation and recording system, new electrode configuration was subsequently designed so we decided to present the latter data.
6-Sep-17	1	Ephys Mapping	Data was captured using a different stimulation and recording system, new electrode configuration was subsequently designed so we decided to present the latter data.
11-Oct-17	1	Ephys Mapping	Data was captured using a different stimulation and recording system, new electrode configuration was subsequently designed so we decided to present the latter data.
7-Nov-17	1	Ephys Mapping	Data was captured using a different stimulation and recording system, new electrode configuration was subsequently designed so we decided to present the latter data.
15-Feb-18	1	Ephys Mapping	Data was captured using a different stimulation and recording system, new electrode configuration was subsequently designed so we decided to present the latter data.
10-Aug-18	1	CT Contrast Test	Performed a high resolution CT scan after 1 week of implant to test whether the electrode could be visualized via MicroCT. The materials of the electrode demonstrated little to no contrast with high resolution CT so the electrode could not be visualized using this technique.
15-Aug-18	1	Material testing/Electroc	Low bending stiffness flexible electrode leads resulted in free movement with less gliosis by GFAP staining than high bending stiffness designs. The electrode lead was carefully explanted to visualize that the lead remained in place on the spinal surface at the targeted level and side of the cord. Data generated used in Figure 2.
8-Aug-19	1	Ephys Mapping	Ephys Mapping. Data generated was presented in Figure 6.
15-Aug-19	1	Ephys Mapping	Ephys Mapping. Data generated was presented in Figures 3, 4, 5 and 6.
11-Sep-19	1	Ephys Mapping	Ephys Mapping. Data generated was presented in Figure 6.
16-Sep-19	1	Ephys Mapping	Ephys Mapping. Data generated was presented in Figure 6.
TOTAL ANIMALS:	26		

Supplementary Table S1: Experiments Summary. Table of all animal experiments performed using the described method with the dates, number of animals, and brief description of the performed procedures.

**Point-to-Reference**	C4	C4	C5	C5	C6	C6	C7	C7	C8	C8	T1	T1
Trapezius	0.3	NS	0.2	0.2	NS	0.2	0.2	0.4	0.3	0.4	NS	NS
Infraspinatus	0.2	NS	0.3	0.2	NS	0.3	0.2	0.2	0.3	0.2	0.3	NS
Triceps	0.5	NS	0.3	0.2	NS	0.3	0.2	0.2	0.3	0.2	0.2	NS
Biceps	0.2	NS	0.3	0.2	NS	0.3	0.3	0.3	0.3	0.5	0.9	NS
W, Flexors	0.2	NS	0.3	0.2	NS	0.3	0.2	0.2	0.2	0.5	0.8	NS
W. Extensors	0.2	NS	0.3	0.3	NS	0.2	0.1	0.2	0.3	0.4	0.7	NS
Hand	0.5	NS	1	0.6	NS	0.4	0.1	0.3	0.3	0.3	0.3	NS

**Point-to-Point**	C4	C4	C5	C5	C6	C6	C7	C7	C8	C8	T1	T1
Trapezius	0.6	0.6	0.6	0.5	0.2	NS	NS	NS	1	0.7	NS	NS
Infraspinatus	0.6	0.5	0.6	0.6	0.4	0.3	0.4	NS	0.8	NS	NS	0.6
Triceps	0.7	0.5	0.6	0.6	0.8	NS	NS	0.9	0.7	0.7	1.2	1
Biceps	0.6	0.5	0.6	0.6	0.8	NS	NS	1	0.8	NS	NS	NS
W. Flexors	0.6	0.5	0.6	0.6	0.4	NS	NS	0.5	0.8	NS	NS	0.8
W. Extensors	0.6	0.6	0.6	0.6	0.8	NS	NS	0.2	0.7	NS	NS	NS
Hand	0.7	0.9	NS	0.7	0.8	NS	NS	0.2	0.9	0.5	NS	0.6

Supplementary Table S2: Thresholds of Modes of Stimulation. Table demonstrating the threshold of activation of each muscle at a given spinal level using point-to-reference and point-to-point stimulation (mA). Thresholds of less than 0.5 mA are highlighted in green to indicate the effective stimulation of a muscle at a given stimulation site. Stimulation sites are presented consecutively from more rostral (left) to caudal (right).

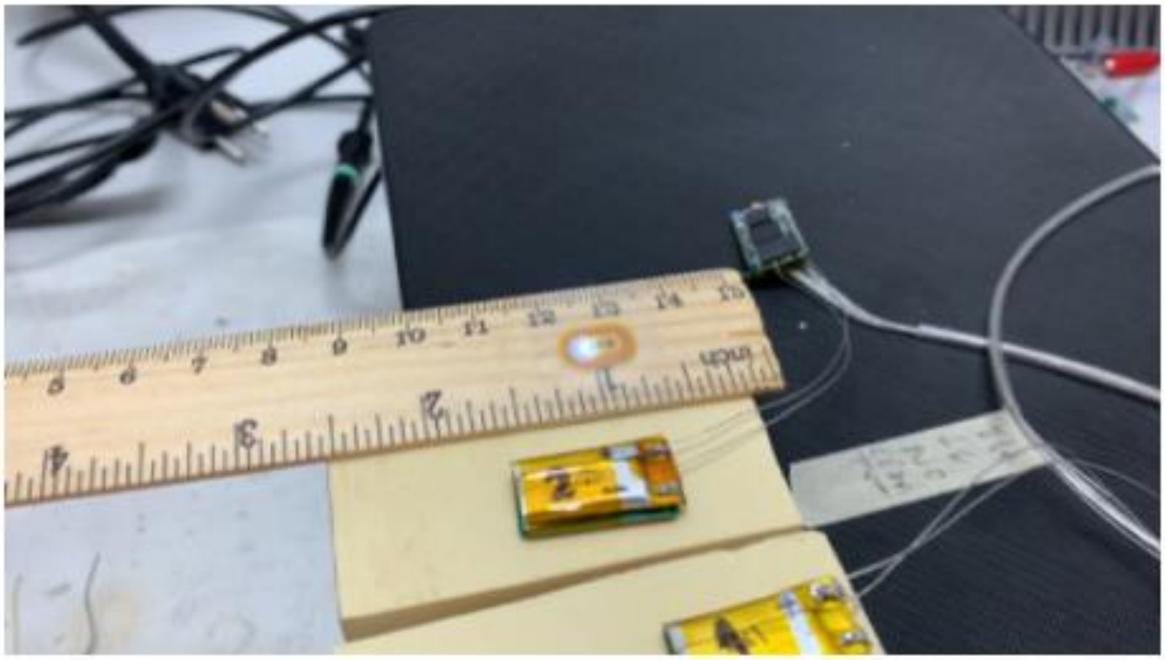

Supplementary Video S1: Wireless Recharging. The video demonstrates a deconstructed spinal stimulation circuit with an inductive charging coil placed on top of an inductive charging pad designed to fit over the bottom of a rat housing chamber. A ruler with an LED powered by an inductive charging coil is also visualized over the charging field. The light demonstrates areas where the battery is effectively able to charge over the surface of the charging pad.

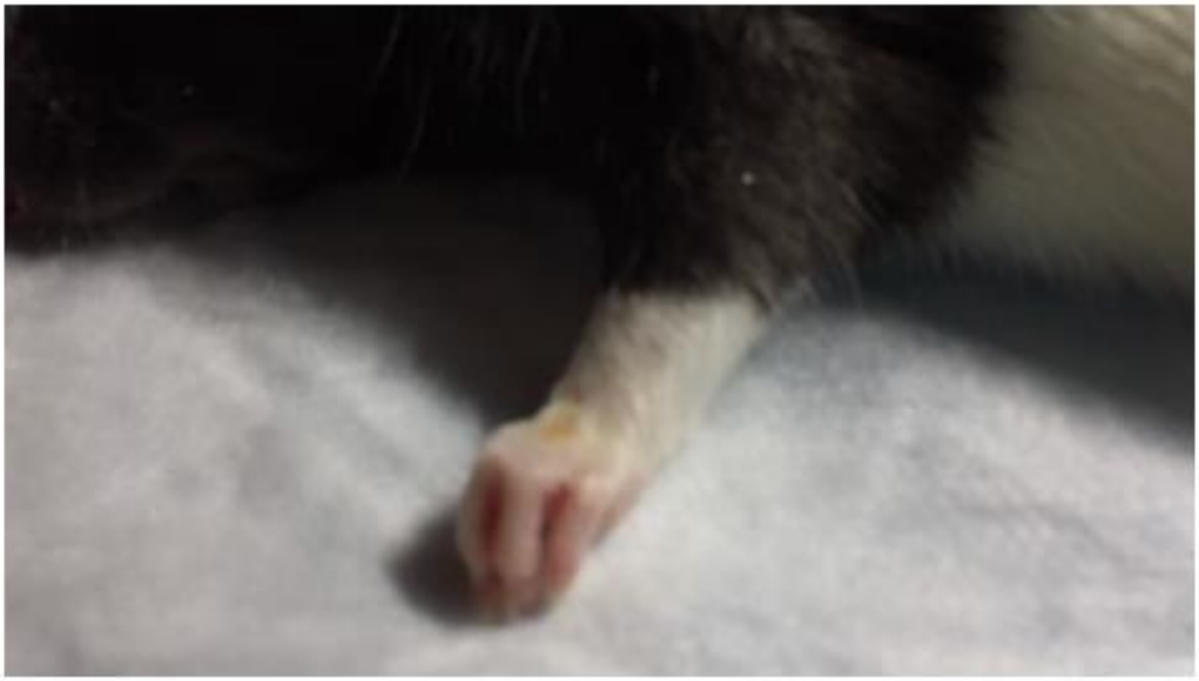

Supplementary Video S2: Selective Fifth Digit Stimulation. The video demonstrates single digit activation during VSS in an anesthetized rat stimulated using point-to-point stimulation at the C7/C8 level.

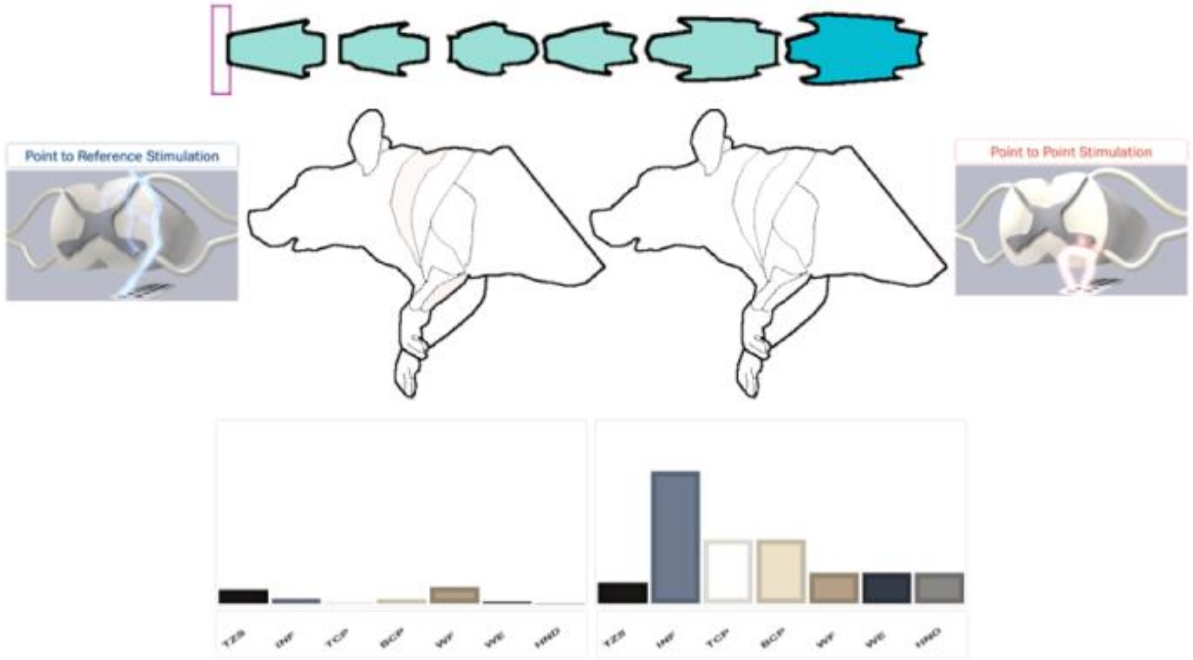

Supplementary Video S3: Video of Spinal Mapping. The video demonstrates the normalized evoked response of each muscle of the forelimb using both point-to-point and point-to-reference stimulation modes along the length of the cervical and upper thoracic spinal cord. The purple box indicates spinal level, and the intensity of red indicates relative muscle activity of a given muscle at a given level. Stimulation was mapped from C4 to T1 spinal levels from left to right.
